# Comparison of Al[^18^F]-NOTA-FAPI-04 PET/CT and [^18^F]-FDG PET/CT in a patient with lung cancer and pulmonary tuberculosis: a case report and literature review

**DOI:** 10.3389/fonc.2025.1470132

**Published:** 2025-02-03

**Authors:** Jingjie Qin, Jinming Yu, Yuchun Wei

**Affiliations:** Department of Radiology, Shandong Cancer Hospital and Institute, Shandong First Medical University, Shandong Academy of Medical Sciences, Jinan, Shandong, China

**Keywords:** FAPI-04, FDG, PET/CT, pulmonary tuberculosis, lung cancer

## Abstract

**Background:**

The coexistence of lung cancer and pulmonary tuberculosis (TB) makes differential diagnosis even more complicated. The purpose of the study is to explore superiority of [^18^F]-NOTA-FAPI-04 PET/CT in distinguishing TB from malignant lesions and accurately detecting inflammatory lymph nodes than [^18^F]-FDG PET/CT.

**Case summary:**

Herein, we described a case report of a patient with both lung cancer and tuberculosis underwent [^18^F]-FDG and Al[^18^F]-NOTA-FAPI-04 positron emission tomography/computed tomography (PET/CT) to determine staging. Additionally, a literature review was conducted to discuss the potential clinical applications of FAPI PET/CT. We reported a 70-year-old man with newly diagnosed lung squamous cell carcinoma underwent [^18^F]-FDG and Al[^18^F]-NOTA-FAPI-04 PET/CT to determine staging. The avid uptake of [^18^F]-FDG in old pulmonary TB and the right hilar inflammatory lymph nodes (<1 cm) were not found on Al [^18^F]-NOTA-FAPI-04 PET/CT. After 2 months of follow-up, the small lymph node was finally confirmed to be inflammatory.

**Conclusion:**

Al[^18^F]-NOTA-FAPI-04 PET/CT may perform better in distinguishing TB from malignancy and may offer greater specificity than [^18^F]-FDG PET/CT for the diagnosis inflammatory lymph nodes.

## 1 Introduction

Lung cancer ranks among the highest in global cancer morbidity and mortality, with non-small cell lung cancer (NSCLC) comprising over 85% of all cases (1, 2). The accurate tumor stage at first diagnosis is crucial to optimize the treatment and improve survival of NSCLC (3). Tuberculosis (TB) is a chronic granulomatosis led by mycobacterium tuberculosis, a public safety problem that endangers global health. Common clinical symptoms of lung cancer and tuberculosis include, but are not limited to, the following: fever, cough, expectoration, hemoptysis, weight loss and anorexia (4–6). When these two diseases coexist or interact, the overlapping imaging and metabolic characteristics complicate diagnosis and treatment.

[^18^F] fluorodeoxyglucose (FDG) is a glucose-based radiotracer whose accumulation correlates with metabolic activity. Positron emission tomography/computed tomography using [^18^F] fluorodeoxyglucose (FDG) is a non-invasive imaging method that has been widely used to distinguish between malignant and benign lesions (**Table 1**). However, [^18^F]-FDG also accumulates in inflammatory cells such as neutrophils, activated macrophages, and lymphocytes at sites of inflammation or infection (11). Therefore, uptake of [^18^F]-FDG has been observed in TB, tuberculoma, and other tuberculosis-associated lesions (12, 13). With PET/CT, both pulmonary and extrapulmonary tuberculosis involvement can be assessed (14). However, it often yields false positives in cases where non-malignant infectious lesions are present (15, 16). SUV_max_, maximum standard uptake value, is a semi-quantitative PET imaging parameter that measures the highest metabolic activity within a lesion. In TB merges malignant lesions, the SUV_max_ tend not to be significantly different (17, 18), which makes the diagnosis specificity of [^18^F]-FDG PET/CT imaging was decreased. Therefore, there is an urgent need for a more specific tracer to accurately stage lung cancer and more clearly distinguish between TB and malignant lesions.

**Table 1:** Comparison of FDG and FAPI PET/CT.

Lesions	FDG PET/CT Finding	FAPI PET/CT Finding	Note
TB	Moderate	Positive	[^68^Ga]Ga-DOTA-FAPI-04 PET/CT showed higher tracer uptake in tuberculous lesions in the lung, spines, and brain than did [^18^F]FDG PET/CT (7).
Intestinal TB	Moderate	Positive	Endoscopy-guided mucosal biopsy of the colon is consistent with tuberculosis, suggesting that ^68^Ga-FAPI may
Matastases,			be valuable in the evaluation of intestinal tuberculosis (8).
mediastinal and hilar lymph nodes	Moderate	Positive	^68^Ga-FAPI PET/CT outperforms ^18^F-FDG PET/CT in the detection of suspected metastases to the brain, lymph nodes, bone, and pleura (9, 10).

FDG, fluorodeoxyglucose; FAPI, fibroblast-activation protein inhibitor; PET, positron emission tomography; TB, tuberculosis.

Fibroblast activation protein (FAP) is a type II membrane-bound glycoprotein from the dipeptidyl peptidase 4 family, highly expressed in cancer-associated fibroblasts (CAFs) and tumor cells of many cancers (19). Recently, radiotracers targeting fibroblast activation protein (FAP) have been developed on account of the FAP-specific inhibitor (FAPI), demonstrating excellent diagnostic performance and offering a novel form of pan-tumor tracing across various cancers (20). Al[^18^F]-NOTA-FAPI PET/CT, using the tracer NOTA (1,4,7-triazacyclononane-N,N’,N’’-triacetic acid) FAPI-04 (Nanchang Tanzhen Biotechnology) was radiolabeled with aluminum fluoride (Al^18^F), is effective for tumor detection (15, 16) and may better facilitate the differentiation of TB from malignancy as well as staging. This interesting case indicates that Al[^18^F]-NOTA-FAPI-04 PET is promising for distinguishing TB from malignancy and may offer more specificity than [^18^F]-FDG for diagnosing inflammatory lymph nodes.

## 2 Case presentation

Our images presented a 70-year-old man with a history of pulmonary tuberculosis and smoking. The patient had no family history of tuberculosis. The patient was admitted to hospital in February 2021 with no apparent cause for sudden syncope and fall, no amaurosis, and unconscious loss. Chest enhanced CT showed a suspicious right lobe mass in the hilum. The patient was subsequently enrolled in a Al[^18^F]-NOTA-FAPI-04 PET/CT clinical trial and underwent both Al[^18^F]-NOTA-FAPI-04 and [^18^F]-FDG PET/CT to clarify the diagnosis. An endoscopic tissue biopsy for diagnostic examination was performed as lung squamous cell carcinoma in the end. Pulmonary TB was diagnosed based on the presence of calcified lesions consistent with prior infection, and no active TB was identified during follow-up testing.

**Figure 1:**
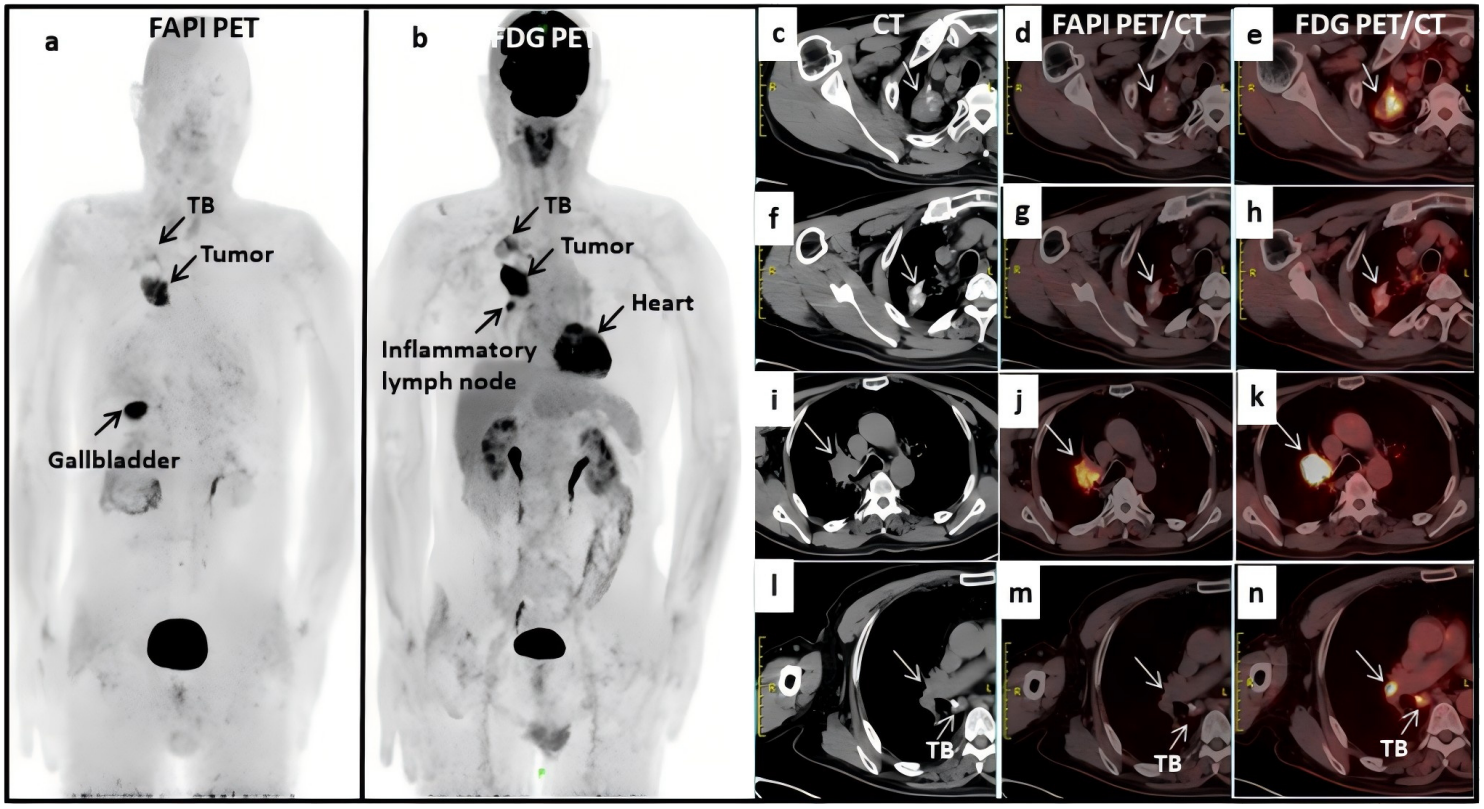
**(A, B)** full body scans with fluorine 18 [^18^F]- labeled fibroblast activation protein inhibitor (FAPI) PET/CT and [^18^F]-labeled fluorodeoxyglucose (FDG); **(C–H)** the old calcified focus and scar tissue of the right lung in CT and FDG PET/CT scans, which were considered as TB; **(I–K)** the metastatic lymph nodes fused with the primary tumor; **(L, M)** the right hilar lymph node was considered an inflammatory lymph node; **(D, G, M)** no avid uptake was observed in Al [^18^F]-NOTA-FAPI-04 PET/CT scans.

Maximum intensity projection images were obtained with both imaging methods (**Figure 1**). Two proficient nuclear medicine physicians reviewed all the images and concurred that the patient had right upper lobe central lung cancer with ipsilateral hilar lymph node metastases, and that the metastatic lymph nodes had fused with the primary tumor (i-k). The old calcified focus and scar tissue of the right lung (c-e, f-h) were considered as pulmonary TB. The right hilar lymph node (l-n) was considered an inflammatory lymph node. The avid uptake of [^18^F]-FDG in old pulmonary TB (e, h) and hilar inflammatory lymph nodes (n) was not observed for Al [^18^F]-NOTA-FAPI-04 (d, g and m). A small inflammatory lymph node in the right hilum (<1 cm) was difficult to distinguish from metastatic lymph nodes, but was finally confirmed on 2-month follow-up. Utilizing dual tracer imaging, the staging was defined to be cT4N1M0, IIIA. The patient subsequently received sequential radiotherapy in April 2021 and chemotherapy in March 2022. The patient’s symptoms improved, and a local tumor recurred *in situ* 1 year later there was no significant adverse effects after treatment. This case indicates that Al[^18^F]-NOTA-FAPI-04 PET/CT is promising for distinguishing TB from malignancy and may offer more specificity than [^18^F]-FDG for diagnosing inflammatory lymph nodes.

## 3 Discussion

### 3.1 FAPI PET/CT has significant advantages over [^18^F]-FDG PET/CT in the diagnosis and accurate staging of lung cancer

The advent of positron emission tomography/computed tomography (PET/CT) has revolutionized the diagnosis and staging of lung cancer (21). [^18^F]-FDG and [^18^F]-FAPI are widely regarded as prominent raidotracers, of which [^18^F]-FDG has showed great advantages in the staging of lung cancer (22, 23). However, emerging evidence suggests that FAPI PET/CT outperforms in the detection and precise staging of lung cancer (24–26).

Wang and Tang et al. reported that [^68^Ga]-FAPI PET/CT is superior to [^18^F]-FDG PET/CT in advanced lung cancer staging, especially in the detection of suspected metastases to brain, lymph nodes (LNs), bone and pleural (9). By targeting FAP, it reduces false positives caused by inflammation and infection and can effectively detect tumors with low metabolic activity (20). Additionally, FAPI has lower uptake in normal tissue (27) and provides excellent imaging contrast, improving the accuracy in identifying small lesions and lymph node metastases in lung cancer.

However, the sensitivity of FDG is finite for some histological types, such as adenocarcinoma *in situ* and several neuroendocrine tumors. [^18^F]-FDG PET/CT is highly sensitive to tumors with high metabolic activity, and it has been deemed to be an effective method for the detection, identification and staging of lung carcinoma (28). However, its specificity has been rigorously challenged for the high uptake of [^18^F]-FDG in both inflammatory and malignant tissues, resulting in false positive diagnoses (29).

Fibroblast activation protein (FAP), a serine protease expressed on cell surface, is highly upregulated in over 90% of epithelial carcinomas (30). FAPI-04, a small molecular inhibitor targeting FAP, has been widely used in PET imaging. Most FAPI-based tracers have been labeled with ^68^Ga, applied in both benign and malignant imaging studies (31). However, owing to its short half-life (T1/2 = 68 min) and short storage time, ^68^Ga is unfit for remote transportation. In comparison, ^18^F (T1/2 = 109 min) is an ideal radionuclide for PET imaging with a high positron yield of 97%, a low mean positron range of 0.5 mm, and no simultaneous γ ray emission (32). However, reports of Al^18^F-labeled FAPI-04 are limited currently. McBride et al. reported an aluminum-fluoride (Al^18^F) chelation strategy in which fluorine is firmly bound to Al^3+^, forming Al^18^F, which is then complexed with NOTA (1,4,7-triazacyclononane-N,N’,N’’-triacetic acid). The complex is subsequently conjugated to the biomolecule of interest (33). The increased prevalence of accelerators in China and the high utilization rate of fluorine standards support the broader adoption of these tracers.

Studies have reported the results of [^18^F]-FAPI PET imaging in a small cohort of patients with lung cancer (34) and breast cancer (35). Wei et al. verified the value of Al[^18^F]-NOTA-FAPI-04 PET/CT for the accurate staging and diagnosis in lung cancer, and they mentioned metastatic LNs with a diameter of 1cm or less are more likely to be omitted than those with a larger diameter, which is even more serious in [^18^F]-FDG PET/CT (10). One reason of false negative results in PET identification of mediastinal lymph node metastases is the poor resolution in identifying microscopic lymph node metastases, a condition referred to as minimal N2 disease due to its relatively favorable prognosis (36). Another reason is that FDG uptake in metastatic lymph nodes cannot be differentiated from uptake in primary tumors or adjacent positive lymph nodes, mainly due to the limited spatial resolution of [^18^F]-FDG PET (37).

In this case report, Al[^18^F]-NOTA-FAPI-04 PET/CT showed greater specificity in distinguishing lung cancer from pulmonary tuberculosis lesions and may diagnose inflammatory lymph nodes more specifically than [^18^F]-FDG. The results of this case further support this perspective and underscore the need for incorporating Al[^18^F]-NOTA-FAPI-04 PET/CT into clinical practice. This imaging modality shows promise in distinguishing tuberculosis from malignant tumors.

### 3.2 The diagnosis function and limitations of [^18^F]-FDG PET/CT in TB

FDG remains the “gold standard” for the detection of high metabolic tumors, especially in rapidly proliferating malignant lesions. Besides, the usefulness of [^18^F]-FDG PET/CT in the management of TB has been investigated (38). Previous studies have shown that FDG has a significant advantage in detecting TB lesions. Since changes in glycolytic activity within the inflammatory foci (as measured by [^18^F]-FDG uptake) correlate well with clinical response markers, [^18^F]-FDG PET/CT imaging may be helpful (39). Firstly, [^18^F]-FDG PET/CT is highly sensitive to metabolically active inflammation and infection, which makes it an effective tool for detecting active TB (40). Due to its high sensitivity, [^18^F]-FDG PET/CT can perform full body scans to detect extra-pulmonary TB spots, allowing for a comprehensive assessment of TB spread and involvement (41). Besides, the ability of [^18^F]-FDG PET/CT distinguishing between active TB and inactive (scar tissue) lesions is crucial in treatment decisions (42). It is also worth mentioning that [^18^F]-FDG PET/CT is capable of monitoring the effect of anti-TB treatment by observing changes in metabolic activity to assess therapeutic response (43).

In active TB granuloma, [^18^F]-FDG accumulates on PET/CT due to the central necrotic nucleus being surrounded by macrophages, epithelioid cells, multinucleated Langerhans giant cells, and lymphocytes, which proliferate and infiltrate in the surrounding normal alveolar space and express glucose carriers (36). Tuberculomas may accumulate [^18^F]-FDG due to increased glucose metabolism caused by active granulomatous inflammation (15). This creates an [^18^F]-FDG signal on PET imaging forming the basis of [^18^F]-FDG-PET imaging in TB (44). However, SUV_max_ is often not significantly different between tuberculosis and malignant lesions (17, 18).

The limitation of [^18^F]-FDG PET/CT for assessing individual lung nodules, especially in endemic areas, is the inability to distinguish between tuberculosis and malignant lesions. [^18^F]-FDG is a non-specific radiotracer accumulated during the inflammation and malignancy; it is not easy to make a diagnosis with [^18^F]-FDG PET/CT only when TB co-exists with lung cancer. Several studies have reported that TB causes false-positive results in patients evaluated for malignant lesions (45, 46). Compared to non-endemic areas, active TB often complicates the diagnosis of lung cancer on [^18^F]-FDG PET/CT, resulting in false positive findings in areas with a high incidence of TB (47). Therefore, identifying a promising alternative radioactive tracer to replace [^18^F]-FDG is crucial for accurate lung cancer staging and differentiation between malignant and tuberculous lesions.

### 3.3 The potential of FAPI PET/CT for accurate diagnosis of TB from the imaging principle and metabolic characteristics

FAPI PET/CT, utilizing tracers such as [^68^Ga]-FAPI and [^18^F]-FAPI, has shown promising advancements in imaging and diagnosing of TB due to its unique targeting mechanism and metabolic profiling capabilities. One of the major strengths of FAPI PET/CT is the high specificity, which makes it a potential tool in the context of complex inflammation. Chen et al. reported that FAPI PET/CT may have a complementary role in reducing false positive results and improving diagnostic accuracy, especially when FDG imaging is unclear (48). Unlike [^18^F]-FDG, which highlights regions of high glucose metabolism often seen in both cancerous and inflammatory conditions, FAPI specifically binds to fibroblast activation protein (FAP) expressed by activated fibroblasts within fibrotic and remodeling tissues (49). Tuberculosis lesions, especially the chronic and fibrotic stages, involve significant fibroblast activity, leading to high uptake of FAPI (7, 8, 50–52). This enables FAPI PET/CT to offer higher specificity, clearer imaging of TB lesions, and effectively highlight TB-related changes. Therefore, FAPI PET/CT may result in fewer false positives and better identify the nature of the TB lesions.

Besides, the reduced background uptake of FAPI PET/CT makes high-contrast images (7). This high contrast is crucial for accurately identifying and delineating TB lesions, providing clear visualizations even in complex anatomical areas. The ability to produce high-resolution images that clarify the boundaries between infected and healthy tissue, improve diagnostic accuracy and facilitate better treatment protocols.

The fast-imaging capability of FAPI PET/CT is another significant advantage. In general, a high-quality image can be obtained within an hour after the tracer is injected (53). This efficiency enhances patient throughput in the clinical settings and increases patient comfort by reducing the scan time.

FAPI reaches a stable physiological distribution in the human body within 10 minutes of injection and maintains this stability for several, providing an extended time window for clinical imaging (19, 54). This makes FAPI PET/CT a potential tool for therapeutic monitoring (55). By evaluating changes in FAP expression before and after treatment, clinicians can monitor the efficacy of anti-TB therapy in real time.

Phlegm coating and molecular diagnosis are still the gold standard of tuberculosis, but functional image technology (such as FDG PET/CT and FAPI PET/CT) has a significant advantage in detecting active lesions and treatment monitoring (56). High-resolution CT (HRCT) provides detailed visualization of lung lesions, including calcifications, fibrosis, cavity formations, and tumor morphology, while MRI offers superior soft tissue resolution, particularly for assessing chest wall and mediastinal involvement (14, 57, 58). However, both techniques rely on morphological features and lack molecular-level functional information, making it challenging to differentiate inflammation from malignancy in cases with overlapping imaging characteristics. Building on the proven success of FDG PET in diagnosing lung tuberculosis, FAPI PET shows promise as a diagnostic tool by specifically targeting areas of fibrosis (42).

Although this study shows the advantages of FAPI PET/CT in the staging of lung cancer and the differentiation of benign and malignant lesions, its limitations should also be of concern. First of all, there is high uptake of FAPI in some benign tumors (such as necrotizing granulomas, renal angiomyolipoma, etc.) (50) and must be interpreted carefully in terms of specificity. In addition, the use of FAPI in certain inflammatory related lesions, such as bronchiolitis obliterans organizing pneumonia, still needs further validation (59).

FDG and FAPI play complementary roles in the diagnosis of lung cancer patients with tuberculosis foci. In this study, we believe that FAPI has certain advantages in tumor staging, but it has not been found that FAPI has significant advantages over FDG in the diagnosis and treatment of tuberculosis. In summary, FAPI PET/CT holds significant potential for improving the staging accuracy in lung cancer and reliability in the diagnosis and treatment of TB, ultimately contributing to better patient management and clinical outcomes.

## 4 Conclusion

The coexistence of lung cancer and pulmonary tuberculosis poses significant challenges for diagnosis and staging of lung cancer, leading to a poor prognosis for patients affected by both diseases. Therefore, accurate estimate and staging at the initial diagnosis are crucial to improve the survival and prognosis of patients. Compared with [^18^F]-FDG, Al[^18^F]-NOTA-FAPI-04 PET/CT has great potential for distinguishing TB from malignant lesions and may offer more specificity than [^18^F]-FDG for diagnosing inflammatory lymph nodes.

## Data Availability

The original contributions presented in the study are included in the article/**Supplementary Material**. Further inquiries can be directed to the corresponding author.

## References

[1] Bray F Laversanne M Sung H Ferlay J Siegel RL Soerjomataram I. Global cancer statistics 2022: GLOBOCAN estimates of incidence and mortality worldwide for 36 cancers in 185 countries. CA Cancer J Clin. (2024) 74(3):229–63. doi: 10.3322/caac.21834 38572751

[2] Miao D Zhao J Han Y Zhou J Li X Zhang T. Management of locally advanced non-small cell lung cancer: State of the art and future directions. Cancer Commun (Lond). (2024) 44:23–46. doi: 10.1002/cac2.12505 37985191 PMC10794016

[3] Herbst RS Morgensztern D Boshoff C. The biology and management of non-small cell lung cancer. Nature. (2018) 553:446–54. doi: 10.1038/nature25183 29364287

[4] Furin J Cox H Pai M. Tuberculosis. Lancet. (2019) 393:1642–56. doi: 10.1016/S0140-6736(19)30308-3 30904262

[5] Bhatt M Kant S Bhaskar R. Pulmonary tuberculosis as differential diagnosis of lung cancer. South Asian J Cancer. (2012) 1:36–42. doi: 10.4103/2278-330X.96507 24455507 PMC3876596

[6] Siracuse CG Litle VR. Identifying lung cancer in patients with active pulmonary tuberculosis. J Thorac Dis. (2018) 10:S3392–7. doi: 10.21037/jtd.2018.07.11 PMC6218365 30505526

[7] Hao B Wu X Pang Y Sun L Wu H Huang W. 18F]FDG and [68Ga]Ga-DOTA-FAPI-04 PET/CT in the evaluation of tuberculous lesions. Eur J Nucl Med Mol Imaging. (2021) 48:651–2. doi: 10.1007/s00259-020-04941-5 32643006

[8] Zheng J Lin K Zheng S Yao S Miao W. 68Ga-FAPI and 18F-PET/CT images in intestinal tuberculosis. Clin Nucl Med. (2022) 47:239–40. doi: 10.1097/RLU.0000000000003917 34619704

[9] Wang L Tang G Hu K Liu X Zhou W Li H. Comparison of 68Ga-FAPI and 18F-FDG PET/CT in the evaluation of advanced lung cancer. Radiology. (2022) 303:191–9. doi: 10.1148/radiol.211424 34981976

[10] Wei Y Ma L Li P Lu J Ren J Yan S. FAPI compared with FDG PET/CT for diagnosis of primary and metastatic lung cancer. Radiology. (2023) 308:e222785. doi: 10.1148/radiol.222785 37552075

[11] Jones HA Clark RJ Rhodes CG Schofield JB Krausz T Haslett C. *In vivo* measurement of neutrophil activity in experimental lung inflammation. Am J Respir Crit Care Med. (1994) 149:1635–9. doi: 10.1164/ajrccm.149.6.7516252 7516252

[12] Kim IJ Lee JS Kim SJ Kim YK Jeong YJ Jun S. Double-phase 18F-FDG PET-CT for determination of pulmonary tuberculoma activity. Eur J Nucl Med Mol Imaging. (2008) 35:808–14. doi: 10.1007/s00259-007-0585-0 18097664

[13] Hahm CR Park HY Jeon K Um SW Suh GY Chung MP. Solitary pulmonary nodules caused by Mycobacterium tuberculosis and Mycobacterium avium complex. Lung. (2010) 188:25–31. doi: 10.1007/s00408-009-9203-1 19956964

[14] Skoura E Zumla A Bomanji J. Imaging in tuberculosis. Int J Infect Dis. (2015) 32:87–93. doi: 10.1016/j.ijid.2014.12.007 25809762

[15] Goo JM Im JG Do KH Yeo JS Seo JB Kim HY. Pulmonary tuberculoma evaluated by means of FDG PET: findings in 10 cases. Radiology. (2000) 216:117–21. doi: 10.1148/radiology.216.1.r00jl19117 10887236

[16] Demura Y Tsuchida T Ishizaki T Mizuno S Totani Y Ameshima S. 18F-FDG accumulation with PET for differentiation between benign and Malignant lesions in the thorax. J Nucl Med. (2003) 44:540–8. 12679397

[17] Sathekge MM Maes A Pottel H Stoltz A van de Wiele C. Dual time-point FDG PET-CT for differentiating benign from Malignant solitary pulmonary nodules in a TB endemic area. S Afr Med J. (2010) 100:598–601. doi: 10.7196/samj.4082 20822650

[18] Chen CJ Lee BF Yao WJ Cheng L Wu PS Chu CL. Dual-phase 18F-FDG PET in the diagnosis of pulmonary nodules with an initial standard uptake value less than 2. 5. AJR Am J Roentgenol. (2008) 191:475–9. doi: 10.2214/AJR.07.3457 18647920

[19] Giesel FL Kratochwil C Lindner T Marschalek MM Loktev A Lehnert W. 68Ga-FAPI PET/CT: biodistribution and preliminary dosimetry estimate of 2 DOTA-containing FAP-targeting agents in patients with various cancers. J Nucl Med. (2019) 60:386–92. doi: 10.2967/jnumed.118.215913f PMC6424229 30072500

[20] Kratochwil C Flechsig P Lindner T. 68Ga-FAPI PET/CT: tracer uptake in 28 different kinds of cancer. J Nucl Med. (2019) 60:801–5. doi: 10.2967/jnumed.119.227967 PMC6581228 30954939

[21] Schlarbaum KE. PET/CT imaging in lung cancer. J Nucl Med Technol. (2024) 52:91–101. doi: 10.2967/jnmt.124.267843 38839112

[22] Antoch G Stattaus J Nemat AT Marnitz S Beyer T Kuehl H. Non-small cell lung cancer: dual-modality PET/CT in preoperative staging. Radiology. (2003) 229:526–33. doi: 10.1148/radiol.2292021598 14512512

[23] Xu G Zhao L He Z. Performance of whole-body PET/CT for the detection of distant Malignancies in various cancers: a systematic review and meta-analysis. J Nucl Med. (2012) 53:1847–54. doi: 10.2967/jnumed.112.105049 23073605

[24] Hathi DK Jones EF. 68Ga FAPI PET/CT: tracer uptake in 28 different kinds of cancer. Radiol Imaging Cancer. (2019) 1:e194003. doi: 10.1148/rycan.2019194003 33778675 PMC7983761

[25] Chen H Pang Y Wu J Zhao L Hao B Wu J. Comparison of [68Ga]Ga-DOTA-FAPI-04 and [18F] FDG PET/CT for the diagnosis of primary and metastatic lesions in patients with various types of cancer. Eur J Nucl Med Mol Imaging. (2020) 47:1820–32. doi: 10.1007/s00259-020-04769-z 32222810

[26] Sun Y Sun Y Li Z Song S Wu K Mao J. 18F-FAPI PET/CT performs better in evaluating mediastinal and hilar lymph nodes in patients with lung cancer: comparison with 18F-FDG PET/CT. Eur J Med Res. (2024) 29:9. doi: 10.1186/s40001-023-01494-9 38173034 PMC10763273

[27] Hamson EJ Keane FM Tholen S Schilling O Gorrell MD. Understanding fibroblast activation protein (FAP): substrates, activities, expression and targeting for cancer therapy. Proteomics Clin Appl. (2014) 8:454–63. doi: 10.1002/prca.201300095 24470260

[28] Sheikhbahaei S Mena E Yanamadala A Reddy S Solnes LB Wachsmann J. The value of FDG PET/CT in treatment response assessment, follow-up, and surveillance of lung cancer. AJR Am J Roentgenol. (2017) 208:420–33. doi: 10.2214/AJR.16.16532 27726427

[29] Shetty N Noronha V Joshi A Rangarajan V Purandare N Mohapatra PR. Diagnostic and treatment dilemma of dual pathology of lung cancer and disseminated tuberculosis. J Clin Oncol. (2014) 32:e7–9. doi: 10.1200/JCO.2012.46.0667 24395843

[30] Loktev A Lindner T Mier W Debus J Altmann A Jäger D. A tumor-imaging method targeting cancer-associated fibroblasts. J Nucl Med. (2018) 59:1423–9. doi: 10.2967/jnumed.118.210435 PMC6126438 29626120

[31] Ruan D Zhao L Cai J Xu W Sun L Li J. Evaluation of FAPI PET imaging in gastric cancer: a systematic review and meta-analysis. Theranostics. (2023) 13:4694–710. doi: 10.7150/thno.88335 PMC10465231 37649615

[32] Fowler JS Ido T. Initial and subsequent approach for the synthesis of 18FDG. Semin Nucl Med. (2002) 32:6–12. doi: 10.1053/snuc.2002.29270 11839070

[33] McBride WJ Sharkey RM Goldenberg DM. Radiofluorination using aluminum-fluoride (Al18F). EJNMMI Res. (2013) 3:36. doi: 10.1186/2191-219X-3-36 23651690 PMC3665500

[34] Lindner T Altmann A Giesel F Kratochwil C Kleist C Krämer S. 18F-labeled tracers targeting fibroblast activation protein. EJNMMI Radiopharm Chem. (2021) 6:26. doi: 10.1186/s41181-021-00144-x 34417894 PMC8380212

[35] Jiang X Wang X Shen T Yao Y Chen M Li Z. FAPI-04 PET/CT using [18F]AlF labeling strategy: automatic synthesis, quality control, and in ivo assessment in patient. Front Oncol. (2021) 11:649148. doi: 10.3389/fonc.2021.649148 33816303 PMC8017320

[36] Schrevens L Lorent N Dooms C Vansteenkiste J. The role of PET scan in diagnosis, staging, and management of non-small cell lung cancer. Oncologist. (2004) 9:633–43. doi: 10.1634/theoncologist.9-6-633 15561807

[37] Li S Zheng Q Ma Y Wang Y Feng Y Zhao B. Implications of false negative and false positive diagnosis in lymph node staging of NSCLC by means of ¹⁸F-FDG PET/CT. PLoS One. (2013) 8:e78552. doi: 10.1371/journal.pone.0078552 24205256 PMC3808350

[38] Sánchez-Montalvá A Barios M Salvador F Villar A Tórtola T Molina-Morant D. Usefulness of FDG PET/CT in the management of tuberculosis. PLoS One. (2019) 14:e0221516. doi: 10.1371/journal.pone.0221516 31454368 PMC6711521

[39] Dureja S Sen IB Acharya S. Potential role of F18 FDG PET-CT as an imaging biomarker for the noninvasive evaluation in uncomplicated skeletal tuberculosis: a prospective clinical observational study. Eur Spine J. (2014) 23:2449–54. doi: 10.1007/s00586-014-3483-8 25070791

[40] Stelzmueller I Huber H Wunn R Hodolic M Mandl M Lamprecht B. 18F-FDG PET/CT in the initial assessment and for follow-up in patients with tuberculosis. Clin Nucl Med. (2016) 41:e187–94. doi: 10.1097/RLU.0000000000001102 26704732

[41] Vorster M Sathekge MM Bomanji J. Advances in imaging of tuberculosis: the role of ¹⁸F-FDG PET and PET/CT. Curr Opin Pulm Med. (2014) 20:287–93. doi: 10.1097/MCP.0000000000000043 24614238

[42] Priftakis D Riaz S Zumla A Bomanji J. Towards more accurate 18F-fluorodeoxyglucose positron emission tomography (¹⁸F-FDG PET) imaging in active and latent tuberculosis. Int J Infect Dis. (2020) 92S:S85–90. doi: 10.1016/j.ijid.2020.02.017 32114199

[43] Yu WY Lu PX Assadi M Huang XL Skrahin A Rosenthal A. Updates on 18F-FDG-PET/CT as a clinical tool for tuberculosis evaluation and therapeutic monitoring. Quant Imaging Med Surg. (2019) 9:1132–46. doi: 10.21037/qims.2019.05.24 PMC6629575 31367568

[44] Hunter RL Actor JK Hwang SA Karev V Jagannath C. Pathogenesis of post primary tuberculosis: immunity and hypersensitivity in the development of cavities. Ann Clin Lab Sci. (2014) 44:365–87. 25361920

[45] Ankrah AO Glaudemans AWJM Maes A Van de Wiele C Dierckx RAJO Vorster M. Tuberculosis. Semin Nucl Med. (2018) 48:108–30. doi: 10.1053/j.semnuclmed.2017.10.005 29452616

[46] Sümbül AT Sezer A Abali H Gültepe B Koçer E Reyhan M. An old enemy not to be forgotten during PET CT scanning of cancer patients: tuberculosis. Contemp Oncol (Pozn). (2016) 20:188–91. doi: 10.5114/wo.2014.43985 PMC4925724 27358601

[47] Akgul AG Liman ST Topcu S Yuksel M. False positive PET scan deserves attention. J BUON. (2014) 19:836–41. 25261676

[48] Chen H Zhao L Ruan D Pang Y Hao B Dai Y. Usefulness of [68Ga]Ga-DOTA-FAPI-04 PET/CT in patients presenting with inconclusive [18F]FDG PET/CT findings. Eur J Nucl Med Mol Imaging. (2021) 48:73–86. doi: 10.1007/s00259-020-04940-6 32588089

[49] Niyonkuru A Bakari KH Lan X. 18F-fluoro-2-deoxy-d-glucose PET/computed tomography evaluation of lung cancer in populations with high prevalence of tuberculosis and other granulomatous disease. PET Clin. (2018) 13:19–31. doi: 10.1016/j.cpet.2017.08.003 29157383

[50] Hotta M Rieger AC Jafarvand MG Menon N Farolfi A Benz MR. Non-oncologic incidental uptake on FAPI PET/CT imaging. Br J Radiol. (1142) 2023:96. doi: 10.1259/bjr.20220463 PMC9975522 35776566

[51] Liu W Gong W Yang X Xu T Chen Y. Increased FAPI activity in pulmonary tuberculosis. Clin Nucl Med. (2023) 48:188–9. doi: 10.1097/RLU.0000000000004498 36607369

[52] Alçın G Tatar G Şahin R Baloğlu MC Çermik TF. Peritoneal tuberculosis mimicking peritoneal carcinomatosis on 68 ga-FAPI-04 and 18 F-FDG PET/CT. Clin Nucl Med. (2022) 47:e557–8. doi: 10.1097/RLU.0000000000004174 35384886

[53] Mori Y Dendl K Cardinale J Kratochwil C Giesel FL Haberkorn U. FAPI PET: fibroblast activation protein inhibitor use in oncologic and nononcologic disease. Radiology. (2023) 306:e220749. doi: 10.1148/radiol.220749 36594838

[54] Hu K Wang L Wu H Huang S Tian Y Wang Q. 18F]FAPI-42 PET imaging in cancer patients: optimal acquisition time, biodistribution, and comparison with [68Ga]Ga-FAPI-04. Eur J Nucl Med Mol Imaging. (2022) 49:2833–43. doi: 10.1007/s00259-021-05646-z 34893920

[55] Wei Y Zheng J Ma L Liu X Xu S Wang S. 18F]AlF-NOTA-FAPI-04: FAP-targeting specificity, biodistribution, and PET/CT imaging of various cancers. Eur J Nucl Med Mol Imaging. (2022) 49:2761–73. doi: 10.1007/s00259-022-05758-0 35262766

[56] Suárez I Fünger SM Kröger S Rademacher J Fätkenheuer G Rybniker J. The diagnosis and treatment of tuberculosis. Dtsch Arztebl Int. (2019) 116:729–35. doi: 10.3238/arztebl.2019.0729 31755407

[57] Lawal IO Abubakar S Ankrah AO Sathekge MM. Molecular imaging of tuberculosis. Semin Nucl Med. (2023) 53:37–56. doi: 10.1053/j.semnuclmed.2022.07.001 35882621

[58] Liang S Xu X Yang Z Du Q Zhou L Shao J. Deep learning for precise diagnosis and subtype triage of drug-resistant tuberculosis on chest computed tomography. MedComm (2020). (2024) 5:e487. doi: 10.1002/mco2.487 38469547 PMC10925488

[59] Shin L Katz DS Yung E. Hypermetabolism on F-18 FDG PET of multiple pulmonary nodules resulting from bronchiolitis obliterans organizing pneumonia. Clin Nucl Med. (2004) 29:654–6. doi: 10.1097/00003072-200410000-00017 15365446

